# Preceding Administration of Minocycline Suppresses Plastic Changes in Cortical Excitatory Propagation in the Model Rat With Partial Infraorbital Nerve Ligation

**DOI:** 10.3389/fneur.2019.01150

**Published:** 2019-11-05

**Authors:** Manabu Zama, Satoshi Fujita, Yuka Nakaya, Morio Tonogi, Masayuki Kobayashi

**Affiliations:** ^1^Department of Pharmacology, Nihon University School of Dentistry, Tokyo, Japan; ^2^Department of Oral and Maxillofacial Surgery, Nihon University School of Dentistry, Tokyo, Japan; ^3^Division of Oral and Craniomaxillofacial Research, Dental Research Center, Nihon University School of Dentistry, Tokyo, Japan; ^4^Molecular Dynamics Imaging Unit, RIKEN Center for Life Science Technologies, Kobe, Japan

**Keywords:** insular cortex, somatosensory cortex, orofacial pain, referred pain, microglia

## Abstract

Neuropathic pain is known to be attributable to the injured nerve, a postoperative problem induced by surgery. The infraorbital nerve (ION), a branch of the trigeminal nerve, innervates to the facial and oral regions and conveys somatosensory information to the central nervous system. The partial ligation of ION (pl-ION) is a method to mimic chronic trigeminal neuropathic pain and behavioral abnormality. To counteract induction of such abnormal pain, the effective pharmacological treatment is desired. Although recent studies have revealed the molecular mechanisms regarding chronic pain, estimation of the effectiveness of the pharmacological treatment has not been well-provided especially in the central nervous system so far. Here we examined whether pl-ION induces plastic changes in the cerebral cortex and investigated effects of minocycline on the cortical plastic changes. We performed the pl-ION to Wistar male rats (4–5 weeks old), and confirmed a mechanical nocifensive behavior in response to the mechanical stimulation with von-Frey filaments. The withdrawal threshold to mechanical stimuli of the whisker pad was decreased 1 day (1 d) after pl-ION, which continued up to 14 d after pl-ION, suggesting that pl-ION model rats presented allodynia and enhanced the response sustained at least for 14 d after pl-ION. Next, cerebrocortical activities were evaluated 3 d after pl-ION (3d-pl-ION) by the optical imaging with a voltages-sensitive dye, RH1691, to quantify the response to electrical stimulation of the whisker pad skin, mandibular molar dental pulp, and mentum skin. Electrical stimulation to the whisker pad skin induced smaller excitation in the primary sensory cortex (S1) of 3d-pl-ION in comparison to that in the sham. In contrast, cerebral cortical responses to the mandibular molar dental pulp and mentum skin stimuli increased both in S1, and the secondary somatosensory and insular oral region (S2/IOR) after pl-ION. Administration of minocycline (30 mg/kg/d) from 1 d before to 2 d after pl-ION partially recovered the pl-ION-induced changes in cortical excitation in S1 and S2/IOR in 3d-pl-ION. These results suggest that somatosensory and insular cortical excitation is changed by pl-ION, and the preceding injection of minocycline counteracts the plastic changes in the cortical activities.

## Introduction

The trigeminal nerve has three major branches, the ophthalmic, maxillary, and mandibular nerves. The infraorbital nerve (ION) is a branch of the maxillary nerve, which innervates to the middle part of the maxillofacial region including the whisker pad. Partial ligation of ION (pl-ION) induces changes in spontaneous behaviors and responses to mechanical stimulation (1, 2). The animal with pl-ION shows lower threshold for a brisk withdrawal of the head responding to mechanical stimulation of the ipsilateral whisker pad, and allodynia starts from 1 day (1 d) after pl-ION and sustains more than 3 weeks (2). Interestingly, allodynia also occurs in the surrounding region of the pl-ION and in the contralateral whisker pad (2), indicating that ectopic pain occurs in the pl-ION model.

The dorsal part of the insular cortex (IC) around the middle cerebral artery (MCA) and the adjacent secondary somatosensory cortex (S2) receive sensory information from the oral structures including the dental pulp (3, 4) and the periodontal ligament (5, 6). The S2 and IC related to the sensation in the oral structure (S2/IOR) project to the limbic structures such as amygdala (7, 8) and lateral hypothalamic area (7). In addition, the recent anatomical studies have demonstrated the descending projections to the trigeminal caudal subnucleus (medullary dorsal horn; Vc), which receives nociceptive inputs from the orofacial structures (9) and transmits these information to the higher brain regions including the parabrachial nucleus and thalamus (10). Therefore, S2/IOR is considered to play a key role in nociceptive information processing of the oral structures (11).

Recent studies have demonstrated that disturbance of nociceptive inputs induces plastic changes not only in the peripheral but also in the central nervous system including the cerebral cortex, and such plastic changes in the cortex may be a part of mechanisms underlying the refractory pain (12). Indeed, trigeminal nerve injury causes plastic changes in neural responses of S2/IOR. We have demonstrated that 1–2 weeks after transection of the inferior alveolar nerve, a branch of the mandibular nerve, S2/IOR shows hyperexcitability responding to electrical stimulation of the upper molar pulp, which is innervated by the maxillary nerve (13). A part of the mechanisms for this plastic change occur in the local circuits of S2/IOR. This finding suggests a possibility that hyperexcitability of S2/IOR may trigger chronic pain even after recovery of the peripheral nervous system that is directly damaged.

Not a few studies in terms of the injury models of the trigeminal nerve have reported the substances that suppress pain-related behaviors. Activation of microglial cells is considered to be a key process that induces neuropathic pain. A variety of cytokines and neurotrophic factors have been reported to induce neuroplastic changes and central sensitization noted above (14, 15). However, little information is available how these drugs affect on neural activities in the cerebral cortex. Taking that the cortical responses reflect integrated information from the peripheral nerve to the cerebral cortex, clarification of drug effects on cortical responses provides critical information of the mechanisms for suppression of nociception.

Minocycline is one of the microglial inhibitors, and the present study aimed to elucidate the effect of minocycline application to the pl-ION model on hyperexcited cortical activities in S1/2 and IC. We performed an *in vivo* optical imaging with RH1691, a voltage-sensitive dye, under urethane anesthesia. The amplitude of optical signals mediated by the voltage-sensitive dye correlates to the membrane potential and reflects excitatory and inhibitory postsynaptic potentials in real time (16), and thus, we can quantify the spatiotemporal patterns of neural excitation with a high resolution. The findings obtained from this study shed light on a new approach to prevent chronic pain induced by peripheral nerve injury.

## Materials and Methods

The Animal Experimentation Committee of Nihon University approved the present experiments, which were performed according to the institutional guidelines for the care and use of experimental animals described in the National Institute of Health *Guide for the Care and Use of Laboratory Animals*. All efforts were made to minimize animal suffering and to reduce the number of animals used.

### Animals

**Figure 1:**
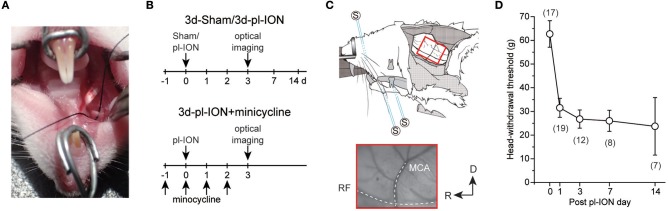
Experimental protocols for partial ligation of the infraorbital nerve (pl-ION) and optical imaging. **(A)** An intraoral approach to expose ION. A black silk thread was placed under ION to show ION clearly. **(B)** Experimental schedules. On 0 d, pl-ION was performed, and a behavioral test was performed 1, 3, 7, and 14 d after pl-ION. Optical imaging was performed 3d after pl-ION. In the group administrated with minocycline, minocycline (30 mg/kg/d) was intraperitoneally injected from 1 d before and 2 d after pl-ION. **(C)** Schematic drawing of the *in vivo* preparation for the optical imaging and position of the stimulation electrodes [modified from (18) with permission]. The lower panel indicate an example of the image of the cortical surface including S1 and S2/IOR. MCA, middle cerebral artery; RF, rhinal fissure. R, rostral; D, dorsal. **(D)** The temporal profile of head-withdrawal threshold (HWT) before and after pl-ION. The number in the parenthesis indicates the number of animals.

Four to 5-week-old male Wistar rats (Sankyo Labo, Tokyo, Japan) were anesthetized with intraperitoneal (i.p.) administration of butorphanol (2.5 mg/kg, Meiji Seika Pharma, Tokyo, Japan), medetomidine (0.375 mg/kg, Zenoaq, Fukushima, Japan), and midazolam (2.0 mg/kg, Sandoz, Tokyo, Japan) dissolved in saline. A pl-ION was performed via the oral cavity (17). Briefly, gingivobuccal fold was incised to expose the ION (Figure 1A). An one-half to one-third thickness of the ION was trapped and ligated tightly by 5–0 silk. The incision was sutured with 5–0 silk. The sham operation was identical except for nerve ligation.

In the model of minocycline treatment, minocycline hydrochloride (30 mg/kg/d, i.p.; Sigma-Aldrich, St Louis, USA) was daily administered from 1 d before pl-ION to 2 d after pl-ION. The 3d-pl-ION received saline injection instead of minocycline.

### Behavioral Test

The head-withdrawal threshold (HWT) to mechanical stimulation of the ipsilateral whisker pad skin was measured using von Frey filaments (4, 8, 15, 26, 30, 40, 50, 60, and 100 g of force; North Coast Medical, Morgan Hill, USA) before and 1, 3, 7, and 14 d after pl-ION (Figure 1B). The animals were restrained around the trunk with a cloth to calm them and were treated gently during the experiments. Training sessions were performed for at least 7 consecutive days. Each stimulus was applied five times in each series of trials. HWT for mechanical stimulation was determined as the minimum pressure intensity that evoked head-withdrawal behavior in response to more than 3 of 5 stimuli. All behavioral tests were performed under blinded conditions.

### *In vivo* Cerebrocortical Imaging

Optical imaging was performed as previously described (3–6, 13, 19–21). Briefly the sham and pl-ION model rats were anesthetized with urethane (1.5 g/kg, i.p.), and then, atropine methyl bromide was administrated (0.5 mg/kg, i.p.). The toe pinch reflex was monitored to check the depth of anesthesia, and additional urethane was administered as needed. Body temperature was kept at 37°C using a rectal probe and heating pad (BWT-100, Bio Research Center, Osaka, Japan). The local anesthetic, lidocaine (2% gel, AstraZeneca, Tokyo, Japan), was administered to the incised skin. The anesthetized rats were fixed to a custom-made stereotaxic snout frame (Narishige, Tokyo, Japan), which was tilted 60° laterally, and a craniotomy was performed (Figure 1C).

RH1691 (1 mg/ml; Optical Imaging, New York) in saline was applied to the cortical surface for 1 h. This application method of RH1691 stains cells between the cortical surface and the deeper layer III. Thus, the optical signals reflect changes in the membrane potential of neurons in layers I–III (3). Changes in RH1691 fluorescence were measured using the CCD camera (MiCAM02, Brainvision, Tokyo, Japan), which was mounted on a stereomicroscope (Leica Microsystems, Wetzlar, Germany). The cortical surface was illuminated through a 632-nm excitation filter and a dichroic mirror using a tungsten-halogen lamp (CLS150XD, Leica Microsystems). The fluorescent emission was captured through an absorption filter (λ > 650-nm longpass, Andover, Salem, MA). The size and resolution of the image captured with the CCD camera was 6.4 × 4.8 mm^2^ and 184 × 124 pixels, respectively.

To remove signals due to acute bleaching of the dye, values without stimuli were subtracted from each recording with stimulation. The sampling interval was 4 ms (250 Hz), and the acquisition time was 500 ms, which was mostly longer than 90–10% decay time of optical signals responding to the stimulation. Forty consecutive images were averaged to improve signal to noise ratio.

### Stimulation of the Skin and Dental Pulps

To quantify the cortical excitation responding to electrical stimulation of the regions innervated by the maxillary and mandibular nerves, we inserted bipolar electrodes, which were made from enamel-coated copper wire (diameter = 80 μm), into the whisker pad skin, mandibular first molar pulp (Figure 1C), and mentum skin (3). Five rectangular voltage pulses (5 V, 100-μs duration, 20 ms interstimulus interval) were applied by a stimulator (STG2008, Multi-Channel Systems, Reutlingen, Germany) at 0.05 Hz to obtain stable cortical responses.

### Data Analysis

The optical imaging data were processed and analyzed using Brain Vision Analyzer software (Brainvision, Tokyo, Japan). Changes in the intensity of fluorescence (ΔF) of each pixel relative to the initial intensity of fluorescence (F) were calculated (ΔF/F), and the ratio was processed with a spatial filter (9 × 9 pixels). A significant response was defined as a signal exceeding seven times the SD of the baseline noise, as previously described (3). Images were aligned across multiple rats using the rhinal fissure and MCA as landmarkers. In a part of animals, the rhinal fissure and the MCA could not be aligned with the other animals due to angioplany; therefore, we excluded the results obtained from these animals. We estimated the spatial profiles of excitation using the initial and maximum responses. The initial response was obtained by outlining the excitation evoked in the first frame that exhibited a significant increase in the optical signal. The maximum response was defined as the outline of the excitatory response in the frame with the maximum amplitude of the optical signal in the center of the initial response. We defined the peak amplitude at the maximum amplitude of an optical response at the point of the initial response.

### Statistics

The data are expressed as the mean ± SEM. Student's *t*-test was used in the analyses. In multiple comparisons, we applied the Bonferroni correction with a Bonferroni-corrected probability value of *P* < 0.016 considered statistically significant.

## Results

### Temporal Changes in HWT of pl-ION Models

Head-withdrawal reflex elicited by mechanical stimulation using von Frey filaments was examined to evaluate nociceptive threshold in pl-ION models. HWT of nociceptive responses to the application of von Frey filaments showed a significant decrease 1–14 d after pl-ION in comparison to that before pl-ION, indicating the development of mechanical hyperalgesia (Figure 1D). Clinically, it is considered that treatments of neuropathic pain are more effective in the earlier period, and thus, we focused on the profile 3 d after pl-ION in the following analyses.

### Cortical Responses to Electrical Stimulation of the Whisker Pad Skin

ION innervates to the whisker pad skin, and pl-ION injured a part of ION. To examine the effect of pl-ION on the cortical activities, we first stimulated the whisker pad skin using a bipolar electrode.

**Figure 2:**
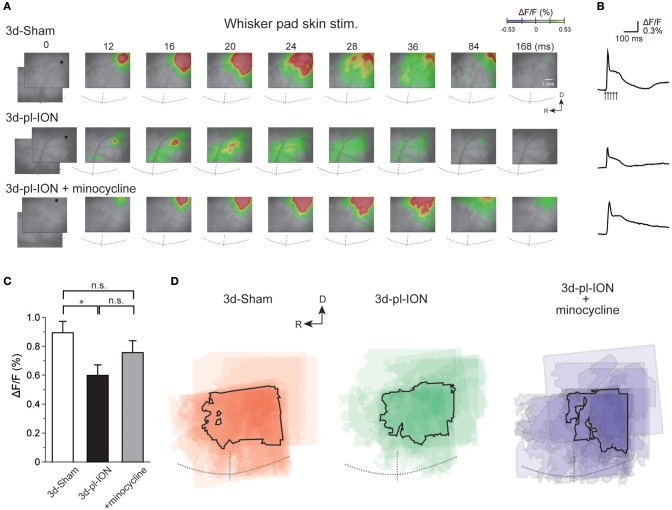
Cortical responses to electrical stimulation of the whisker pad skin. **(A)** Representative examples of cortical responses in 3 d after sham operation (3d-Sham), 3 d after pl-ION (3d-pl-ION), and 3d-pl-ION that received minocycline administration (3d-pl-ION+minocycline). The time from the onset of the electrical stimulation of the whisker pad skin is shown on the top of each panel. R, rostral; D, dorsal. **(B)** Temporal profiles of optical signals in the regions of interest (ROIs; black circles) in S1/S2 shown in **(A)**. **(C)** The amplitude of S1/S2 excitation in 3d-Sham (*N* = 18), 3d-pl-ION (*N* = 13), and 3d-pl-ION with minocycline (*N* = 15). **(D)** Superimposed images of the maximum responses evoked by the whisker pad skin stimulation. The number of overlapping responses is represented by gradation of the color density. The line outlines the area responding to stimulation in 50% of animals. *P < 0.016, Student's *t*-test with Bonferroni correction.

The responses were consistently observed in the barrel field that involves both S1 and S2 responding to electrical stimuli at 5 V (Figures 2A,B) as previously reported (3, 22). The excitation in S1 and S2 frequently expanded beyond the field of view, and thus we quantified only the amplitude of excitation in the initially evoked response region. Because of the difficulty in identifying the border between S1 and S2, we quantified the amplitude in the initial region of excitation as the representative amplitude in S1/S2. For the comparison to the excited regions responding to whisker pad skin stimulation, we first imaged IC around MCA, which corresponds to IOR, and then moved the field of view dorsally to image the barrel cortex.

The peak amplitudes of cortical excitation in S1/S2 of 3d-Sham were 0.89 ± 0.08% (*N* = 18). Cortical excitation 3 d after pl-ION was significantly reduced to 0.60 ± 0.07% (*N* = 13; *P* < 0.016, Student's *t*-test with Bonferroni correction; Figure 2C). There was little difference in the evoked S1/S2 regions between 3d-Sham and 3d-pl-ION as shown in Figure 2D. These results suggest that pl-ION decreases somatosensory sensation 3d after the treatment.

### Cortical Responses to Electrical Stimulation of the Mandibular Molar Pulp

The mandibular first molar pulp is innervated by the inferior alveolar nerve, a branch of the mandibular nerve, which is adjacent to the maxillary nerve but is not injured in the present experimental condition. To test whether pl-ION induces ectopic pain as previously reported in similar nerve injury models (13, 23), we examined the profiles of cortical responses to the mandibular molar pulp stimulation.

**Figure 3:**
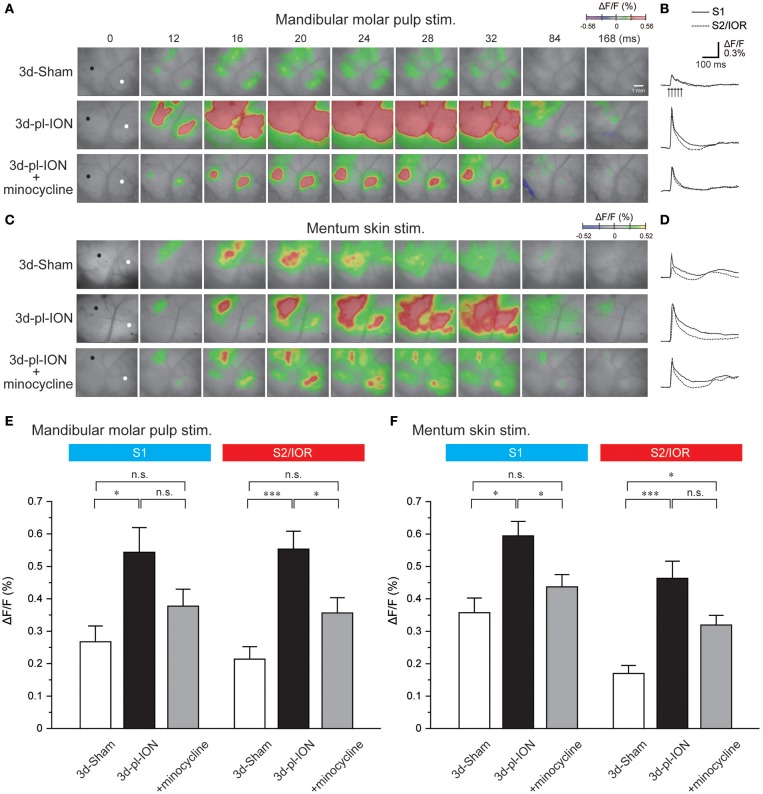
Cortical responses to electrical stimulation of the mandibular molar pulp and mentum skin. **(A)** Representative examples of cortical responses in 3d-Sham, 3d-pl-ION, and 3d-pl-ION+minocycline to mandibular molar pulp stimulation. The time from the onset of the electrical stimulation of the whisker pad skin is shown on the top of each panel. **(B)** Temporal profiles of optical signals in the ROIs: black circles in S1 (thick lines) and white circles in S2/IOR (dotted lines) shown in **(A)**. **(C)** Representative examples of cortical responses in 3d-Sham, 3d-pl-ION, and 3d-pl-ION+minocycline to mentum skin stimulation. **(D)** Temporal profiles of optical signals in the ROIs: black circles in S1 (thick lines) and white circles in S2/IOR (dotted lines) shown in **(C)**. **(E)** The amplitude of S1 (blue) and S2/IOR (red) excitation in 3d-Sham (*N* = 13), 3d-pl-ION (*N* = 13), and 3d-pl-ION with minocycline (*N* = 14) in response to stimulation of the mandibular molar pulp. **(F)** The amplitude of S1 (blue) and S2/IOR (red) excitation in 3d-Sham (*N* = 7), 3d-pl-ION (*N* = 15), and 3d-pl-ION with minocycline (*N* = 15) in response to stimulation of the mentum skin. *P < 0.016, ***P < 0.001, n.s., not significant (Student's *t*-test with Bonferroni correction).

The initial responses to electrical stimulation of the mandibular molar pulp were detected in two cortical regions: the rostroventral part of S1 and dorsocaudal part in reference to the cross point of the rhinal fissure and MCA (Figures 3A,B). The latter corresponds to S2/IOR as previously we reported (3, 4). Excitation spread to the surrounding cortical regions in a concentric manner. Similar to the case of the whisker pad skin stimulation, we stimulated the molar pulps at 5 V because the responses in S1 and S2/IOR was consistently observed at 5 V (3).

In 3d-Sham, the peak amplitudes of cortical excitation in S1 and S2/IOR were 0.27 ± 0.05% (*N* = 13) and 0.21 ± 0.04% (*N* = 13), respectively. Cortical excitation 3 d after pl-ION was facilitated in both S1 and S2/IOR (Figure 3E). In 3d-pl-ION, the peak amplitude of cortical excitation in S1 was significantly larger than that in 3d-Sham (0.54 ± 0.08%, *N* = 13; *P* < 0.016, Student's *t*-test with Bonferroni correction). Similarly, S2/IOR showed larger cortical excitation in 3d-pl-ION than that of the Sham (0.55 ± 0.06%, *N* = 13; *P* < 0.001, Student's *t*-test with Bonferroni correction). These results suggest that ectopic pain occurs in 3d-pl-ION.

### Cortical Responses to Electrical Stimulation of the Mentum Skin

The skin is consistently stimulated, whereas the dental pulps are protected from mechanical stimulation by the dentin and enamel. These differences might cause a distinct effect of pl-ION on evoked cortical responses between the skin and dental pulps, although stimulation of the dental pulps by the bipolar electrodes has an advantage to restrict the stimulated region. We, therefore, examined the cortical responses to stimulating the mentum skin, which is innervated by the mental nerve, a branch of the mandibular nerve.

In 3d-Sham, the peak amplitudes of cortical excitation in S1 and S2/IOR were 0.36 ± 0.05% (*N* = 7) and 0.17 ± 0.07% (*N* = 7), respectively. Cortical excitation 3 d after pl-ION was facilitated in both S1 and S2/IOR (Figures 3C,D,F). In 3d-pl-ION, the peak amplitude of cortical excitation in S1 was significantly larger than that in 3d-Sham (0.59 ± 0.04%, *N* = 15; *P* < 0.016, Student's *t*-test with Bonferroni correction). Similarly, S2/IOR in 3d-pl-ION showed larger cortical responses to the mentum skin stimulation than that in 3d-Sham (0.46 ± 0.05%, *N* = 15; *P* < 0.001, Student's *t*-test with Bonferroni correction). These results suggest ectopic pain occurs 3d-pl-ION that not only in the dental pulp but also in the skin.

### Minocycline Partially Recovers Abnormal Cortical Responses to Electrical Stimulation of the Orofacial Regions

In the experiment of whisker pad stimulation, administration of minocycline (30 mg/kg; i.p.) once a day from a day before and 0, 1, and 2 d after pl-ION (Figure 1B) tended to recover pl-ION-induced change in the amplitude of the cortical excitation in S1/S2 to 3d-Sham though the difference was not significant (0.76 ± 0.08%, *N* = 15; *P* = 0.17, Student's *t*-test with Bonferroni correction; Figure 2). Indeed, no significant difference was detected in the comparison to 3d-Sham and to 3d-pl-ION with minocycline (*P* = 0.24, Student's *t*-test with Bonferroni correction).

On the other hand, minocycline decreased pl-ION-induced hyperexcitation of cortical responses to mandibular molar pulp and mentum skin stimulation (Figure 3). In minocycline-administered animals, mandibular molar pulp stimulation induced comparable cortical excitation in S1 compared to that of 3d-Sham (0.38 ± 0.05%, *N* = 14; *P* = 0.15, Student's *t*-test with Bonferroni correction), though this amplitude was not different from that in 3d-pl-ION (*P* = 0.09, Student's *t*-test with Bonferroni correction). In S2/IOR, the group of 3d-pl-ION with minocycline showed a tendency of slightly higher amplitude of cortical excitation (0.36 ± 0.05%, *N* = 14) to that in 3d-Sham (*P* = 0.03, Student's *t*-test with Bonferroni correction), and this amplitude was significantly smaller than that in 3d-pl-ION (*P* < 0.016, Student's *t*-test with Bonferroni correction).

In terms of mentum skin stimulation, minocycline suppressed the pl-ION-induced increase of cortical responses in S1 (0.44 ± 0.04%, *N* = 15; *P* < 0.016; Student's *t*-test with Bonferroni correction). In addition, there was no significant difference in the amplitude between 3d-Sham and 3d-pl-ION + minocycline (*P* = 0.18, Student's *t*-test with Bonferroni correction). On the other hand, in the group of 3d-pl-ION with minocycline, excitation in S2/IOR (0.32 ± 0.03%, *N* = 15) had a tendency of smaller amplitude of cortical excitation to that in 3d-pl-ION (*P* = 0.05, Student's *t*-test with Bonferroni correction), though this amplitude was significantly larger than that in 3d-Sham (*P* < 0.016, Student's *t*-test with Bonferroni correction).

These results suggest that minocycline suppresses pl-ION-induced facilitative neural activities, which may be a part of mechanism for inducing ectopic pain.

### Effects of pl-ION and Minocycline on the Area of Cortical Excitation

**Figure 4:**
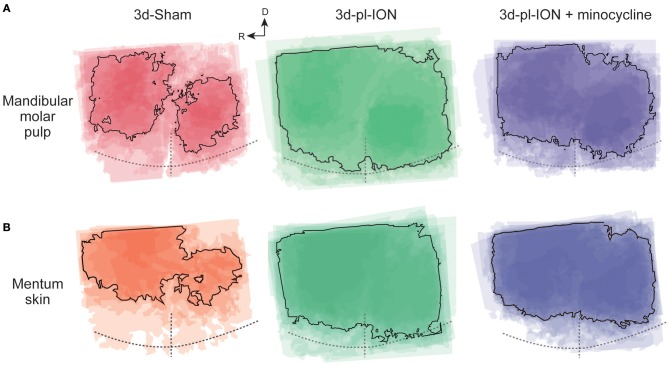
Spatial profile of cortical responses to electrical stimulation of the mandibular molar pulp and mentum skin. **(A,B)** Superimposed images of the maximum responses evoked by the mandibular molar pulp stimulation **(A)** and mentum skin stimulation **(B)** in 3d-Sham, 3d-pl-ION, and 3d-pl-ION+minocycline. R, rostral; D, dorsal.

Accompanied with the analysis of the amplitude of cortical excitation responding to orofacial stimulation, the area of excitation was analyzed in the Sham, pl-ION model, and minocycline-administered model. Although the excited area should be quantified by measuring the pixels whose signals exceeded the threshold, the cortical excitation frequently propagated to the dorsal S1 and S2 especially in 3d-pl-ION (Figure 4). Therefore, the accurate quantification of the excited area could not be done in the present study. Alternatively, the areas of excitation were merged at the timing when the excitation amplitude reached the peak, and we specified the outline of the excited areas overlapped in ≥50% animals.

In term of the stimulation of uninjured branches of mandibular nerves, the area analysis showed the similar tendency, i.e., a facilitative effect of pl-ION and a suppressive effect of minocycline in consistent with the analysis of the amplitude (Figure 4). In 3d-pl-ION, the excited area responding to electrical stimulation of the mandibular molar pulp and mentum skin expanded especially toward the dorsal region, which corresponds to S1 and S2. On the other hand, minocycline administration reduced the facilitative expansion of excitatory area by pl-ION (Figure 4), which corresponded to the analysis of the amplitude of the cortical excitation.

## Discussion

The present study demonstrated the plastic changes in the spatiotemporal excitation patterns in S1/2 and IC of the model with pl-ION. The response to the whisker pad skin stimulation, which activates regions innervated by injured ION, was suppressed, whereas S1/2 and IC responses were facilitated to the stimulation of the mandibular molar pulp and mentum skin, which are innervated by adjacent branches of the maxillary nerve including ION. These changes are considered to underlie the ectopic pain induced by pl-ION. Administration of minocycline partially inhibited these cortical changes, suggesting that suppression of microglial activation may be effective to suppress ectopic pain induction.

### Hypoexcitation in S1/S2 Responding to Whisker Pad Skin Stimulation

According to our previous study, electrical stimulation of the whisker pad skin in control rats induced excitation rather in the dorsal region compared to the cortical responses to the mandibular molar pulp and mentum skin (3) (Figures 2–4). This area corresponds to the barrel cortex, which is clearly presented by flat mount sections (3). It is known that whisker sensation is processed in both S1 and S2 with the barrel structure in layer IV (24), and the somatotopic map showed these fields symmetrically organized. Therefore, it is difficult to discriminate whether the excited region is located in S1 or S2 by the view of the cortical surface, and we did not discriminate between S1 and S2 excited regions in the experiment of stimulating the whisker pad stimulation. While several recordings showed a clear excitation in S2/IOR responding to whisker pad skin stimulation, most cases showed a faint activation in S2/IOR, and thus, we did not analyzed the excitation kinetics in S2/IOR.

The treatment of pl-ION injures a part of nerve fibers of ION, and therefore, it is reasonable that 3d-pl-ION showed lower excitation in S1/S2 responding to whisker pad stimulation. However, behavioral studies including the present study have demonstrated the hyperexcitability of neural activities in the primary and secondary neurons (25, 26). This seems to contradict to the result of suppressive cortical excitation in 3d-pl-ION. A possible explanation for the discrepancy is that pl-ION increases spontaneous neural activities of the secondary neurons in Vc (27), and these activities may induce continuous excitation of the cortical neurons. Indeed, Latremoliere et al. (14) reported an increase in spontaneous behaviors correlating to pain: an increment of face-grooming activity. If this is the case, the present optical imaging technique cannot estimate the increase in the baseline activities, because we quantified the differences between evoked and baseline signals as mentioned in the Materials and Methods. This discrepancy should be further explored by another method such as *in vivo* Ca^2+^ imaging.

### Hyperexcitation in S1 and S2/IOR Responding to Mandibular Dental Pulp and Mentum Skin Stimulation

In contrast to the responses to the whisker pad skin stimulation, mandibular molar pulp stimulation and mentum skin stimulation in pl-ION models induced larger excitatory responses in S1 and S2/IOR than those in controls. Because S1 exhibits a clear somatotopy, the expanded excitatory propagation in S1 is a possible underlying mechanism for the enlargement of the receptive field in the orofacial area and lower capacity to detect the stimulated region. In addition, larger response in S1 may contribute to hypersensitivity responding to mechanical sensation.

On the other hand, S2/IOR is likely to detect nociception rather than touch (11). For example, electrical and mechanical stimuli of the periodontal ligaments induce excitation principally in S2/IOR and S1, respectively (5, 19). According to this idea, larger excitation in S2/IOR may reflect higher sensitivity to nociceptive inputs, which would cause hyperalgesia and allodynia. These results are consistent with behavioral findings of the decrease in HWT reported in this and previous studies (17, 28).

The mechanisms for abnormal pain induced by pl-ION have been explored. Shinoda et al. (17) demonstrated an involvement of P2X_3_ receptors expressed in the trigeminal ganglion neurons. Several neurochemical marker expressions including calcitonin gene-related peptide and substance P, and NK1 receptors are also changed in Vc, where the secondary neurons exist (2). In contrast, little information has been obtained with respect to the contribution of higher brain regions to abnormal pain induced by pl-ION. We consider that not only the primary and secondary neurons but also the cerebrocortical neurons and their local circuits may be changed by pl-ION, because the transection model of the inferior alveolar nerve shows hyperexcitation, which accompanies plastic changes in excitatory and inhibitory synaptic transmission in the cortical circuits (13).

### Minocycline Relieves Trigeminal Neuropathic Pain

Minocycline is a popular tetracycline, which inhibits protein synthesis of bacteria by a blockade of tRNA binding to ribosomal subunit (30S). In the nervous system, minocycline is known to inhibit activation of microglia, and cytokines and other bioactive substances have been reported to be involved in modulation of microglial activities: interleukin (IL)-1, IL-1β, IL-6, nitric oxide, prostaglandin, and so on (14, 29, 30).

It has been explored whether minocycline administration relieves neuropathic pain that occurs in the orofacial regions innervated by the trigeminal nerve (14, 23, 31). The inferior alveolar nerve and mental nerve transection model that shows hypersensitivity estimated by mechanical stimulation of the whisker pad show minocycline-dependent suppression of hypersensitivity possibly by inhibiting p38 mitogen-activated protein kinase in microglia of Vc (31). Shibuta et al. (23) demonstrated that minocycline suppresses activation of microglia in parallel to the attenuation of Vc neuronal activities in the pl-ION model showing mechanical allodynia. The present results that minocycline partially recovers cortical activity changes induced by pl-ION corroborate these previous reports.

We consider that inhibition of microglial activation before pl-ION is critical in minocycline-induced recovery of cortical response changes by pl-ION. Our pilot study suggests that the effect of minocycline application started just after ION and sequential application once a day (1 mg/kg) had little effect on cortical responses. This issue should be further examined in the future.

## Data Availability Statement

All datasets generated for this study are included in the article.

## Ethics Statement

The animal study was reviewed and approved by The Animal Experimentation Committee of Nihon University.

## Author Contributions

MK designed the research. MZ performed the research. SF and MZ analyzed the data. MZ, YN, MT, and MK wrote the paper.

### Conflict of Interest

The authors declare that the research was conducted in the absence of any commercial or financial relationships that could be construed as a potential conflict of interest.
